# Correlating Histological Results and Total Serum Levels of the Prostate‐Specific Antigen Among Patients in Southwestern Uganda

**DOI:** 10.1155/proc/9924021

**Published:** 2026-02-24

**Authors:** Saphurah Nabaasa, Marvin Mwesigwa Mutakooha, Lawrence Amadile, Charles Nkubi Bagenda, Jolly Lydia Ninsiima, Abraham Birungi, Raymond Atwine, Hassan Wasswa, Richard Kasadha, Tibenderana Lauben, Frank Ssedyabane

**Affiliations:** ^1^ Department of Medical Laboratory Sciences, Faculty of Medicine, Mbarara University of Science and Technology, Mbarara, P.O. Box 1410, Uganda, must.ac.ug; ^2^ Department of Surgery, Faculty of Medicine, Mbarara University of Science and Technology, Mbarara, P. O. Box 1410, Uganda, must.ac.ug; ^3^ Department of Medical Laboratory Sciences, Faculty of Health Science, Muni University, Arua, P.O. Box 725, Uganda, muni.ac.ug; ^4^ Department of Pathology, Faculty of Medicine, Mbarara University of Science and Technology, Mbarara, P.O Box 1410, Uganda, must.ac.ug; ^5^ Uganda Cancer Institute, Division of Pathology and Laboratory Medicine, Mulago Hill Road, Kampala, P.O BOX 3935, Uganda, uci.or.ug; ^6^ Mayanja Memorial Medical Training Institute, School of Medical Laboratory Technology, Mbarara, P.O BOX 348, Uganda

**Keywords:** benign prostate hyperplasia, histological findings, prostate adenocarcinoma, prostate intraepithelial neoplasia, prostate-specific antigen

## Abstract

**Background:**

Both healthy and malignant prostate tissues express the glycoprotein marker known as prostate‐specific antigen (PSA). When checking for prostate lesions, serum total PSA levels are a major factor. However, the exact levels to rely on are not explicit.

**Objective:**

To ascertain the relationship between histopathological findings and serum levels of the PSA in patients in southwest Uganda.

**Methods:**

This cross‐sectional study involved 71 participants in southwestern Uganda from January to July 2023, who underwent histological examinations. Blood samples were taken off for total serum PSA level measurement. Stained formalin‐fixed paraffin‐embedded sections were examined. Histological results and PSA levels were correlated using Spearman’s correlation coefficient.

**Results:**

The study involved 74 participants with an average age of 74.20 ± 9.40 years and average Gleason score of 7.73 ± 1.04. Only 1/71 (1.41%) had prostatic intraepithelial neoplasia (PIN), 36/71 (50.70%) had benign prostate hyperplasia (BPH), and 34/71 (47.89%) had prostate adenocarcinoma (PAC). A significant correlation was observed between PSA levels above 100 ng/mL (*p* = 0.001, rho = 0.5955) and prostate cancer and between PSA levels up to 20 ng/mL (*p* = 0.010, rho = 0.03033). AUC of 0.85 (95% CI: 0.77–0.94) showed good predictive power of the test. PSA optimal cut off was 103.4 ng/mL, at sensitivity of 68% and specificity of 92% with maximum Youden index (J): 0.595.

**Conclusion:**

There was a significant correlation between BPH with PSA levels up to 20 ng/mL and above 100 ng/mL for prostate adenocarcinoma. In some of the cases, however, total serum PSA levels were high for BPH and low for prostate adenocarcinoma. PSA test usefulness cannot be nullified, but its accuracy and specificity have to be ascertained in order to increase its reliability. Future researches are argued to focus more on how to refine PSA‐based diagnostics through identifying any underlying unknown hereditary factors and probably better biomarkers that could be influencing PSA levels. With this, dependability increases and unnecessary biopsing reduces, thus alleviating anxiety in patients and probably their caregivers.

**Contributions of this study:**

The study provides additional insights into the importance and clarity of total serum PSA levels in prostate screening and diagnosis.

## 1. Introduction

The prostate is a small organ in the body that is composed of muscular and glandular tissues surrounding a part of the urethra in men and is in part responsible for producing fluid for semen [1]. From the GLOBOCAN cancer statistics 2022, prostate cancer (Pca) ranks third among cancers disturbing men worldwide with a percentage of 7.3% after lung and colorectal cancers (with 23.4% and 9.6%, respectively) [2].

The prostate can be affected by a number of benign and malignant tumors. The most frequent malignant condition is Pca, which can cause major health problems such as bone pain, spinal cord compression, lower urinary tract symptoms, and side effects from vigorous treatment [3–5]. Drawing from the results of a retrospective analysis of 874 patient medical records pertaining to Pca diagnoses made between January 1, 2015, and December 31, 2019, of the patients with comorbidities, 154 (17.62%) had hypertension, 33 (3.78%) had heart disease, 56 (6.4%) had diabetes mellitus, and 49 (5.6%) had HIV. Among additional symptoms, lower urinary tract symptoms accounted for 306 (35.01%) of all presenting complaints, with bone pain coming in second at 60 (6.86%) [3]. Pca is the second most common cancer among men in Uganda, with an incidence of 6.4% in 2018, which included over 2086 new cases and 1177 deaths [6]. In one of the studies in Uganda, Pca incidence was higher with age‐standardized incidence rate (ASR) of 55.1 per 100,000 person‐years (PY) followed by Kaposi sarcoma with 27.7 [7]. There is an estimation of this cancer incidence projecting from 41.6–60.5/100,000 PY [8] which calls for more attention in sensitization and early screening.

The same way all cancers have risk factors, Pca is not exceptional; there are multiple factors that increase the risk of a person developing it. Several of them have been identified in previous researches. Some risks that are based on diet, lifestyle, and occupation have been found not to be strongly associated and others are undoubtedly associated with Pca development [9]. Some of these include age, men who are 50 years and above stand a higher risk [9, 10]. It was also noted men from African decency also had higher chances of developing this cancer and increased deaths from it being more prevalent in African American compared to the White men [10]. It has also been noted that Pca could be running in genes; men who have had their close relatives suffer from it stand higher chances of developing it than those who have never had any family member suffer from this cancer [9]. The more we get exposed to the knowledge about risk factors of these cancers, the more we learn how to maneuver well when it comes to prevention, screening, diagnosis, and effective treatment.

Benign prostatic hyperplasia (BPH) is the most common type of benign lesion [11]. BPH itself is characterized by progressive enlargement of the prostate, urination difficulties, and an increase in serum PSA with the commonest complications being urinary infections, bladder stone accumulation, and chronic and acute urine retention [12, 13]. Other disorders include prostatitis and prostatic intraepithelial neoplasia (PIN).

Proliferation and anaplasia of the cells lining the prostatic ducts, ductules, and acini are hallmarks of PIN, which is thought to be a probable precursor lesion of invasive Pca. However, unlike prostate carcinoma, the impact of PIN on the serum prostate‐specific antigen (PSA) concentration is controversial [14]. Clinically, these lesions present with similar symptoms as mentioned earlier [15]. Pca may be specifically associated with anemia, weight loss, and lower back pain, especially if metastatic [16].

A Gleason score is typically used to rate Pca based on the distinction of malignant cells from healthy cells. A Gleason score of six or lower might be referred to as low‐grade or well‐differentiated. Cancers classified as intermediate‐grade or moderately differentiated may have a Gleason score of 7. One could refer to malignancies with a Gleason score of 8–10 as differentiated cancers [17].

The diagnosis of prostate lesions requires clinical history, physical examination, and biochemical investigations. Clinically, it might be difficult to differentiate lesions because of the similarities in symptom presentation in patients as reported in several studies [18]. Physical examination using digital rectal examination is also subjective and has a very low sensitivity and specificity when used alone [19]. These investigations are usually complemented by testing for serum total PSA levels, a protein secreted by the prostate that is found in both normal and abnormal prostate conditions [20]. PSA is a glycoprotein secreted by prostate gland, with its production increased in both malignant and benign prostate conditions [21]. As a blood test, PSA has the potential to detect prostate lesions in their early stages; however, its purpose and specificity in predicting prostate disorders are subjects of debate [22].

At Mbarara Regional Referral Hospital (MRRH) which serves populations from resource‐limited settings, PSA testing is especially valuable due to its affordability. It is considered a crucial tool and should discreetly expand the capacity to distinguish between BPH and cancer of the prostate [23]. Despite its wide spread usage, serum PSA has notable limitations. One primary concern is that PSA levels are not always straightforward indicators of specific prostate conditions. Although PSA is organ‐specific and highly sensitive, it exhibits low specificity where elevated levels can be seen in benign conditions such as BPH, inflammation, and following diagnostic or surgical procedures [24]. For instance, a PSA level of *e* ≥ 20 ng/mL increases the likelihood of Pca diagnosis via biopsy; however, there are cases of BPH with PSA levels above this threshold complicating interpretation [25].

Currently many hospitals in the region consider 4 ng/mL as the upper limit of normal PSA levels but this cutoff has been shown to have a very low specificity for various prostatic lesions [26]. Despite this, it is generally accepted that higher PSA levels correlate with an increased risk of malignancy [27]. All this can lead to misdiagnosis or overtreatment [24]. This shows the medical fraternity that there are no justifiable serum total PSA levels in regard to prediction of prostatic lesions.

This study aims at generating local data that can be compared to global findings, thereby assessing the relevancy of PSA testing in early diagnosis of prostatic lesions to prevent overtreatment and guide resource‐appropriate screening strategies in settings like MRRH where advanced diagnostics such as MRI or biopsy may be limited. Ultimately, the goal is to bridge the gap between availability of resources and having effective clinical practice, acknowledging PSA’s role within the broader context of Pca diagnosis in resource‐constrained environments.

## 2. Materials and Methods

### 2.1. Ethics Considerations

Ethical approval to conduct this study was obtained from Mbarara University of Science and Technology Research Ethics Committee (MUST‐2022‐714), Faculty Research Committee and Medical Laboratory Sciences Department. The Administration of MRRH granted administrative authorization to perform this study in the urology unit. Prior to data collection, participants were thoroughly informed about the exact purpose of the study, the procedures involved, and any potential risks. This was clearly done verbally by well‐trained research assistants in the participants’ local languages (Runyankore/Rukiga) for better comprehension. Participants were given a chance to ask questions for clarity and were also informed that they had all the rights to withdraw from the study at any time they felt uncomfortable without any repercussions. Each participant then provided a written informed consent. In a way of protecting confidentiality, all data collection instruments used codes rather than names and only the research team could access the data. This was done for security purposes.

### 2.2. Study Setting

The research was carried out from January to July 2023 at the MRRH’s Urology Department in Mbarara city, Southwest Uganda. About 286 km southwest of Kampala, Uganda’s capital, MRRH is situated in Mbarara city along the Mbarara–Kabale route. It is located close to Mbarara University of Science and Technology, where it serves as a teaching hospital. It has a bed capacity of 350 beds and serves the catchment districts of Ibanda, Rubirizi, Rwampara, Isingiro, Kazo, Bushenyi, Mitooma, Ntungamo, Mbarara, Kiruhura, Sheema, and Buhweju and populations from countries such as Congo, Rwanda, Burundi, and Tanzania. Urology services are available to patients Monday through Sunday at the MRRH’s fully operational inpatient and outpatient units with a bed capacity of nine beds in the urology inpatient unit.

### 2.3. Study Design

A descriptive cross‐sectional design was employed in this investigation. Participants were enrolled using the consecutive sampling strategy based on their urology clinic enrollment as long as their clinical evaluation necessitated a prostate biopsy for histological analysis. Buderer’s method for specificity with a power of 90% was used to get the required sample size of 71 participants [28].

### 2.4. Inclusion Criteria

Participants were included basing on their baseline PSA results and DRE findings. MRRH being a referral hospital, most participants were referred in whose PSA was tested and found to be > 4 ng/mL which is the common cut off in most facilities. Upon arrival, their DREs were taken by the hospital urologists and only those that required a biopsy examination and consented were now included in our study.

### 2.5. Exclusion Criteria

Participants were removed if their biopsies showed no signs of glandular components at all, since they were deemed insufficient for examination and requested a follow‐up biopsy 6 months later. To maintain the sample size, a new participant was enlisted for each removed individual. In order to prevent the impact of prostate manipulation on PSA levels, blood samples were taken from the participants prior to the removal of their prostate biopsies. Prior to their arrival for the PSA measurement, all participants were requested to refrain from sexual activity for a minimum of 48 h. Control for total PSA levels in blood in respect to when the last ejaculation took place was limited because it was based on the word‐of‐mouth from the participants. Formalin‐fixed paraffin‐embedded sections were stained with hematoxylin and eosin staining technique [29] and the stained slides were examined by qualified histopathologists.

### 2.6. Data Collection

#### 2.6.1. Demographic Data Collection

Data on demographics were collected using a pretested semistructured questionnaire administered by the corresponding author or a research assistant (clinic nurse). The semistructured questionnaire was written in the common native language (Runyankore) and English. This gave all our participants a chance to comprehend the context well and freely give their responses. Age, comorbidities, a family history of Pca, and other details were among the information gathered, as seen in Table 1.

**TABLE 1:** Participants’ demographics.

Variable	Category	Frequency	Percentage (%)
Age (years) Mean ± SD 74.20 ± 9.40	< 60	3	4.23
	60–69	19	26.76
	70–79	31	43.66
	> 80	18	25.35

Gleason score (*n* = 34) Mean ± SD 7.73 ± 1.04	≤ 6	4	11.76
	7 (3 + 4)	2	5.88
	7 (4 + 3)	9	26.47
	8	10	29.41
	9 and 10	9	26.47

Districts of residence	Mbarara	11	16.42
	Ntungamo	08	11.94
	Isingiro	06	8.96
	Rukungiri	06	8.96
	Others	40	53.72

Comorbidities	HIV/AIDS	1	1.41
	HTN	4	5.63
	HTN/dementia	1	1.41
	HTN/diabetes	1	1.41
	None	64	90.14

*Note:* This table shows the participants’ demographics. The mean age was 74.2 years (SD0 ± 9.4), with the largest proportion aged 70–79 years (43.66%). The Gleason scores among 34 patients with prostate cancer showed a mean of 7.73 (SD ± 1.04), with distribution ranging from ≤ 6 to 10, indicating varied tumor aggressiveness. Participants resided in diverse districts. Regarding comorbidities, most participants (90.14%) had no reported health conditions, while a small percentage had HIV/AIDS, hypertension, or both. HTN, hypertension. For Gleason score, only adenocarcinoma patients were considered.

Abbreviations: AIDS, acquired immunodeficiency syndrome; HIV, human immunodeficiency virus; SD, standard deviation.

#### 2.6.2. Blood Collection and Prostate‐Specific Antigen Measurements

Blood samples for testing total PSA levels were collected from all consenting patients regardless of their documented baseline PSA results. This was performed to ensure that all serum total PSA measurements were performed using the same machine to minimize discrepancies. Blood was collected at least seven days after each patient’s clinical assessment to avoid falsely raised total PSA results due to DRE [30]. Aseptic venepuncture was used to extract 4 mL of venous blood from the mid‐cubital vein into a standard vacutainer [31]. Identification codes were used on the specimen as labels. Blood samples were allowed to coagulate by standing at room temperature. Following coagulation, the blood components were separated by centrifuging samples at 5000 revolutions per minute and serum transferred to cryovial tubes using a micropipette. The total PSA levels were quantitatively measured using a well‐calibrated MAGLUMI fully‐auto chemiluminescence immunoassay machine (model: Maglumi 2000). Standard operating protocols were followed for running the samples, and all controls were operational. Although controls were performed every day to determine the accuracy of our data, machine calibration was always performed after 14 days. Because of its features that increase its sensitivity and specificity, this machine produces reliable results [32].

#### 2.6.3. Biopsy Collection for Histology and Examination

A urologist surgeon, a senior house officer, a medical officer, and an on‐duty nurse gathered the biopsies. On average, five prostate biopsies were taken from each patient. To avoid cold ischemia, these five biopsies on a single cassette were promptly fixed in 10% formalin. Biopsies were routinely processed to wax using an automatic Histokinnette tissue processing machine Leica brand Tp1020 model in the Histopathology Laboratory at MRRH by a laboratory technologist. To view histological details, 5 mµ sections stained with hematoxylin and eosin dye were placed on glass microscope slides and examined [29].

#### 2.6.4. Hematoxylin and Eosin

Following a 10 min staining process with Harris hematoxylin, the histology sections were differentiated in 1% acid alcohol, rinsed in water, and then blued in running tap water for five minutes. After a minute of eosin Y staining, the sections were quickly dehydrated in 95% alcohol and then three changes of 100% alcohol to achieve total dehydration. Following two xylene changes, the pieces were mounted using DPX.

**FIGURE 1:**
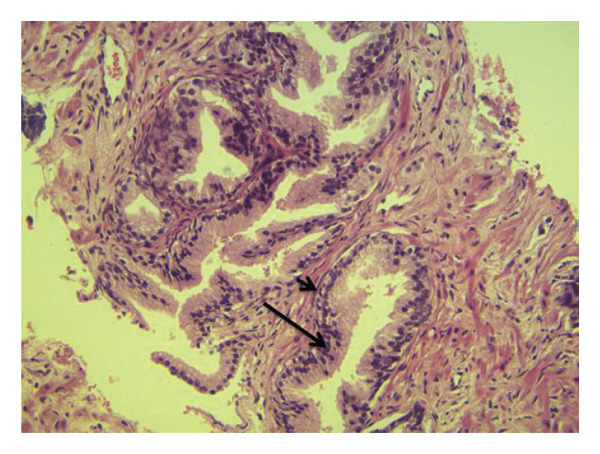
Microscopic histological examination of benign prostate hyperplasia (BPH). Demonstrated are increased cell numbers, variably sized prostatic glands with cytologically bland epithelial cells (long arrow), glandular and stromal cellular proliferation, and the presence of intact basal layer (short arrow) (hematoxylin and eosin stain, x200).

**FIGURE 2:**
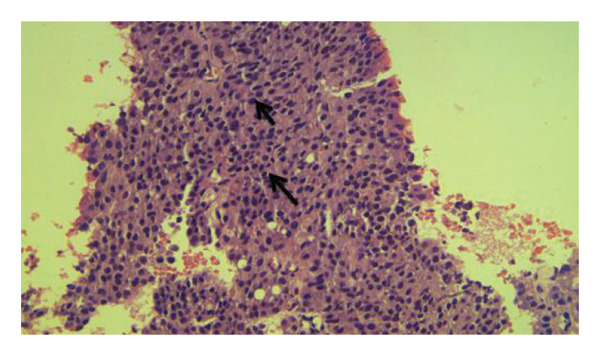
Microscopic histological examination of a prostate tissue showing prostate adenocarcinoma. Demonstrated is the infiltration by sheets of neoplastic epithelial cells with hyperchromatic enlarged nuclei with no glandular differentiation (both arrows) (Gleason score 5 + 5 = 10/10) (hematoxylin and eosin stain, x200).

**FIGURE 3:**
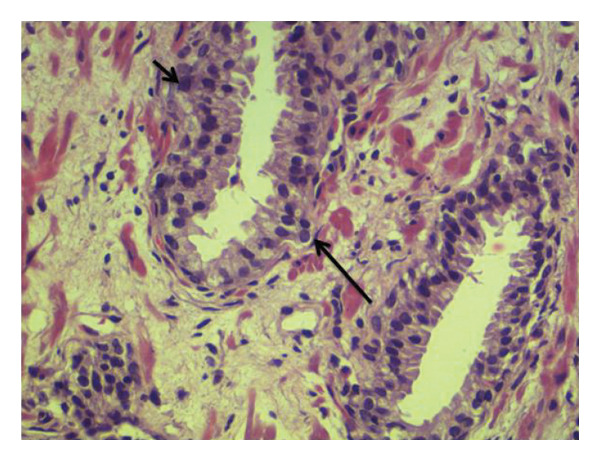
Microscopic histological examination showing low‐grade PIN. Presence of pseudostratification with some enlarged hyperchromatic nuclei. Nucleoli is inconspicuous and the basal layer intact (hematoxylin and eosin stain, x400).

By means of conventional light microscopy, a qualified histopathologist examined the stained slides. Microscopic histological examination for BPH demonstrated increased cell numbers, variably sized prostatic glands with cytologically bland epithelial cells, glandular and stromal cellular proliferation, and the presence of basal layer. Microscopic histological examination for adenocarcinoma demonstrated infiltration by sheets of neoplastic epithelial cells with hyperchromatic enlarged nuclei with no glandular differentiation for PIN, presence of pseudostratification with some enlarged hyperchromatic nuclei with an inconspicuous nucleoli, and intact basal layer (Figures 1, 2, and 3).

Grading of cancer in tissues was based on Gleason grade score on a scale of 6–10 with 6 (3 + 3), 7 (4 + 3 and 3 + 4), 8 (4 + 4), 9 (5 + 4), and 10 (5 + 5). Since all the five biopsies were processed in one cassette, one score that was most predominant was given for all the five biopsies.

#### 2.6.5. Data Management and Analysis

Data were imported into STATA 17 software (StataCorp LLC, College Station, Texas, United States) after being entered into an Excel spread sheet (Microsoft Office Professional Plus 2010). The population was characterized using descriptive statistics using frequencies and means ± standard deviations (SDs), and Spearman’s rank correlation statistical test was used to correlate total PSA levels in blood (categorized in ranges) with histological findings at a significance level of 0.05. Spearman’s rank correlation statistical test was employed since we were working with non‐normally distributed PSA level data. To evaluate the predictive ability of PSA as a continuous variable for identification of binary outcome in histological findings (cancer or noncancer), a receiver operating characteristic (ROC) curve analysis was done. The area under the curve (AUC) computed assisted in determining the diagnostic performance of PSA. A better predictive power is shown by an AUC value that is close to 1.0. A predictive performance is considered statistically significant if the 95% CI for the AUC does not include the null value of 0.5. To appreciate the AUC interpretation better, the AUC of 0.90–1.00 is excellent; 0.80–0.89 is good; 0.70–0.7 is fair; 0.60–0.6 is poor; and 0.50–0.59 is considered a failure [33]. A diagnostic test performance analysis was also done to obtain the sensitivity, specificity, positive predictive value (PPV), and negative predictive value (NPV) of PSA at different thresholds.

## 3. Results

The participants’ average age was 74.20 ± 9.40 years, and their average Gleason score was 7.73 ± 1.04. Most cancer cases had a Gleason score more than 7 (Table 1).

### 3.1. Histological Findings of Trucut Biopsy Specimens

Most of the 71 patients in our study had BPH (36/71, 50.70%), followed by prostate adenocarcinoma (34/71, 47.89%), and one individual had a diagnosis of low‐grade PIN (LGPIN) (1/71, 1.41%) as seen in Table 2. All the 34 Pca cases that were recorded were all adenocarcinomas. The Gleason scores of four individuals were 6, 11 had 7 with two having (3 + 4), nine were with (4 + 3), 10 had 8, and nine had 9‐10, as seen in Table 1. The correlation of PSA levels and Gleason score among participants with Pca was Pearson’s correlation co‐efficient = 0.3959, *p*‐value 0.0226.

**TABLE 2:** Distribution of histological findings from prostate biopsy examination.

Histological findings	Frequency (percentage)	95% CI
BPH	36 (50.70)	39.05–62.28
PIN	1 (1.41)	0.19–9.62
Prostate cancer	34 (47.89)	36.39–59.61
Total	**71**	

*Note:* This table shows the distribution of different histological diagnoses among 71 prostate biopsy samples where benign prostate hyperplasia (BPH) was the predominant histological finding followed by prostate cancer and lastly PIN (prostate intraepithelial neoplasia) which was rarely observed. The bold value represents the total prostate biopsy samples for BPH, PIN, and prostate cancer.

### 3.2. Prostate‐Specific Antigen Level Distribution

**FIGURE 4:**
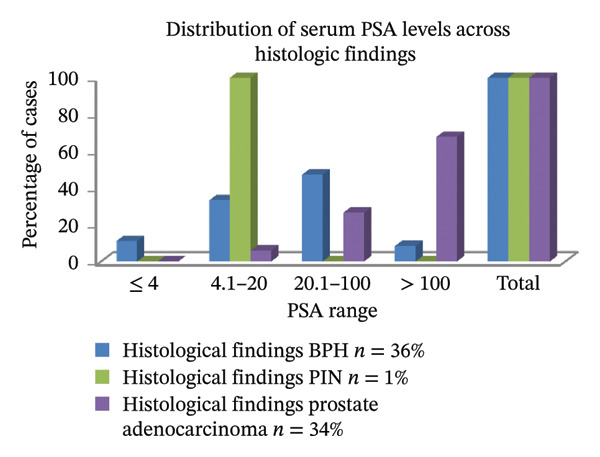
A column chart illustrating the percentage of cases within each PSA range for BPH, PIN, and prostate adenocarcinoma.

Of the 36 BPH subjects, 17 (47.22%) had PSA between 20.1 and 100 ng/mL. The total serum PSA level of the only PIN participant ranged from 4.1 to 20 ng/mL. PSA values of greater than 100 ng/mL were present in more than half of the subjects with Pca 23/34 (67.65%). 3/36 (8.33%) patients had PSA > 100 ng/mL, while 4/36 (11.11%) cases of BPH had PSA < 4 ng/mL (Table 3 and Figure 4).

**TABLE 3:** Distribution of prostate‐specific antigen levels across histological findings.

Serum PSA levels	Histological findings		
	BPH (*n* = 36), *f* (%)	PIN (*n* = 1), *f* (%)	Prostate cancer (*n* = 34), *f* (%)
≤ 4	4 (11.11)	0 (0.00)	0 (00.00)
4.1–20	12 (33.33)	1 (100.00)	2 (5.88)
20.1–100	17 (47.22)	0 (0.00)	9 (26.47)
> 100	3 (8.33)	0 (0.00)	23 (67.65)
Total	36 (100.00)	1 (100.00)	34 (100.00)

*Note:* This table illustrates the distribution (*f* (%), percentage frequency) of PSA (prostate‐specific antigen) levels across different histological conditions: PIN, prostate intraepithelial neoplasia; BPH, benign prostate hyperplasia; and prostate adenocarcinoma. Most BPH cases exhibited PSA levels between 20.1 and 100 ng/mL, while the majority of prostate adenocarcinoma cases had PSA levels exceeding 100 ng/mL, indicating a strong association between elevated PSA and malignancy.

Table 3 and Figure 4 show that the distribution of serum PSA levels varied among different histological groups examined. Almost half of BPH cases (47.2%) exhibited PSA levels greater than 100 ng/mL, with a smaller proportion showing PSA levels ≤ 4 ng/mL (11.1%). Prostate adenocarcinoma cases demonstrated a more diverse PSA distribution, with 26.5% falling within the 20.1–100 ng/mL range and 67.6% exceeding 100 ng/mL. 100% of all PIN cases predominantly had PSA levels between 4.1 and 20 ng/mL. These finding suggest that higher PSA levels are more strongly associated with malignant conditions, while moderate elevations may be indicative of premalignant or benign processes.

### 3.3. Correlation of Prostate‐Specific Antigen Levels With Histological Findings

PIN and PSA levels did not significantly correlate, according to the Spearman’s correlation coefficient. Total serum PSA levels in the ranges of ≤ 4 (*p* = 0.043, rho = 0.2409) and 4.1–20 ng/mL (*p* = 0.010, rho = 0.3033) were statistically significant as correlated with BPH. PSA levels > 100 ng/mL (*p* value 0.001, rho −0.5955) had a negative statistical significance with BPH. The ranges of ≤ 4 ng/mL (*p* = 0.043, rho = −0.2409) and 4.1–20 ng/mL (*p* = 0.010, rho = −0.3033) showed a statistically significant negative correlation with Pca, while levels > 100 ng/mL showed a favorable correlation (*p* = 0.001, rho = 0.5955) (Table 4).

**TABLE 4:** Correlation between prostate‐specific antigen and histological findings.

PSA levels (ng/mL)	PIN, Spearman’s rho (prob > t)	BPH, Spearman’s rho (prob > t)	Prostate adenocarcinoma, Spearman’s rho (prob > *t*)
≤ 4	−0.0292 (0.809)	0.2409 (**0.043**)^∗^	−0.2409 (**0.043**)^∗^
4.1–20	0.2309 (0.053)	0.3033 (**0.010**)^∗^	−0.3033 (**0.010**)^∗^
20.1–100	−0.0909 (0.451)	0.2232 (0.061)	−0.2232 (0.061)
> 100	−0.0909 (0.451)	−0.5955 (**0.001**)^∗^	0.5955 (**0.001**)^∗^

*Note:* This table shows how PSA levels are variably associated with histological findings: BPH, benign prostate hyperplasia; PIN, prostate intraepithelial neoplasia; and prostate adenocarcinoma. Using Spearman’s rho coefficient, the strengths and direction of correlations are shown across different PSA ranges. Statistically significant values (*p* < 0.05) are highlighted.

^∗^Statistically significant values (*p* < 0.05) are highlighted.

**FIGURE 5:**
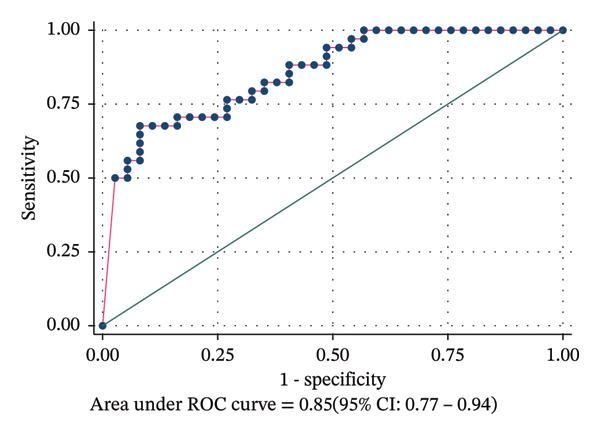
Receiver operating characteristic (ROC) curve showing predictive performance of PSA for prostate adenocarcinoma. At optimal cut off of 103.4, sensitivity of 68%, and specificity of 92% and with Youden index (J): 0.595, PSA had a significant and good predictive power; the AUC: 0.85 (95% CI: 0.77 0.94) was obtained to discriminate participants with prostate adenocarcinoma from those without.

Predictive ability of PSAA ROC curve analysis generated an AUC of 0.85 (95% CI: 0.77–0.94). PSA optimal cut off was 103.4 ng/mL, sensitivity was 68%, and specificity was 92% with Youden index (J): 0.595; PSA had a significant and good predictive power to discriminate participants with prostate adenocarcinoma from those without cancer, as seen in Figure 5.

### 3.4. Evaluation of PSA Levels at Different Thresholds for Pca Diagnosis

At PSA 4, sensitivity was 100%; % CI: 100–100; specificity was 10.81%; % CI: 3.59–18.03; PPV was 50.75%; % CI: 39.12–62.38; and NPV was 100%; % CI: 100–100.

At PSA10, sensitivity was 100%; % CI: 100–100; specificity was 35.14%; % CI: 24.03–46.24; PPV was 58.62%; % CI: 47.16–70.08; and NPV was 100%; % CI: 100–100.

At PSA20, sensitivity was 94.12%; % CI: 88.64–99.59, specificity was 45.95%; % CI: 34.35–57.54; PPV was 61.54%; % CI: 50.22–72.85; and NPV was 89.47%; % CI: 82.34–96.61.

At PSA100, sensitivity was 67.65%; % CI: 56.77–78.53; specificity was 91.89%; % CI: 85.54–98.24; PPV was 88.03%; % CI: 81.03–95.89; and NPV was 75.56%; % CI: 65.56–85.55, as presented in Table 5.

**TABLE 5:** Evaluation of PSA levels at different thresholds for prostate cancer diagnosis.

PSA cut‐off point (ng/mL)	Gold standard test	Diagnostic accuracy metrics			Gold standard test
	Absent, *N* = 37	Present, *N* = 34	Metric in %	95% CI	Absent, *N* = 37
PSA4			Sens	100	100–100
Absent	4	0	Spec	10.81	3.59–18.03
Present	33	34	PPV	50.75	39.12–62.38
			NPV	100	100–100

PSA10			Sens	100	100–100
Absent	13	0	Spec	35.14	24.03–46.24
Present	24	34	PPV	58.62	47.16–70.08
			NPV	100	100–100

PSA20			Sens	94.12	88.64–99.59
Absent	17	2	Spec	45.95	34.35–57.54
Present	20	32	PPV	61.54	50.22–72.85
			NPV	89.47	82.34–96.61

PSA100			Sens	67.65	56.77–78.53
Absent	34	11	Spec	91.89	85.54–98.24
Present	3	23	PPV	88.03	81.03–95.89
			NPV	75.56	65.56–85.55

*Note:* This table demonstrates how accurately prostate cancer can be predicted at different PSA thresholds (4, 10, 20, and 100 ng/mL). As PSA levels go up, the likelihood of cancer also increases. For lower PSA levels (4 ng/mL), most cases are captured (100% sensitivity) but many are not cancers (PPV = 50.75%) and this threshold is also very good at ruling out cancer when levels are below 4 (NPV = 100%). As the PSA thresholds increase, sensitivity decreases slightly, but the ability to correctly identify those with or without cancer (specificity) improves. At PSA levels of 100 ng/mL, less sensitive (67.65%) but very specific (91.89%), there is a high probability (88.03%) of having prostate cancer. This indicates that very high PSA levels are highly predictive, but lower thresholds catch more cases early enough.

## 4. Discussion

Thirty six/71 (50.70%) of the individuals had BPH, 34/71 (47.89%) had Pca, and only one (1.41%) had PIN. This observation is in agreement with several other studies [11, 34–36]. All of these studies identified a higher number of BPH cases, followed by prostate adenocarcinoma. The current study’s findings were not the same as those of Goeman et al., [37] which reported a very high number of participants with LGPIN, 43/104 (41.3%), compared with one participant that had LGPIN 1/71 (1.41%). This may be because their investigation used a variety of techniques to get prostate tissue, such as trucut biopsies, transurethral resection of the prostate (TURP), and radical prostatectomy, whereas the current study solely used trucut biopsies. The present study results do not agree with Nzeyimana et al., where the majority, 100/114 (87.7%), had Pca and a few, 14/114 (12.3%), had BPH [38].

All 34 (100%) of the cancer patients in this study were found to be adenocarcinomas. This result was comparable to Belbase et al., [39], and Oluwole et al., [40]. Our present study did not agree with several other studies that identified different types of Pca. For instance, Hirachand et al. [36] had some small cell carcinomas (SCC) and Samad et al. [35] had some squamous, large, and transitional cell carcinomas. Similarly, Pudasaini et al. [34] reported some sarcomatoid carcinomas. In addition, Fatima et al. [17] identified acinar and ductal carcinomas in Pca. However, it is readily apparent that the most prevalent kind of Pca in males is always adenocarcinoma [41].

In the present study, most participants with Pca had a Gleason score above seven [34, 39], and only 4/34 had a Gleason score of 6. The current study’s results were consistent with Pudasaini et al. [34] and Okolo et al. [42] who reported similar findings with the majority of participants with cancer with Gleason score > 7 peaking at 9‐10. The results of the current investigation, however, contradicted Josephine [43] and Lokuhetty et al. [44] where the Gleason scores of most cancer patients ranged from 2 to 4. This similarity could be attributed to the cancer stage and presence or absence of metastasis. Our study identified a significant correlation between Gleason score and PSA levels among participants with prostate adenocarcinoma whereby the cancer patients that had higher levels of PSA also had higher Gleason scores (rho 0.3959, *p* value = 0.0226) consistent with a previous study done in Tanzania (*p* value = 0.005) [45] and Nigeria [42]; and Lojanapiwat et al. though reported it to be average because a few patients had born metastasis showing advanced cancer but with low PSA levels [25]. Our results were inconsistent with previous literature in one study by Kouji et al. in Japan where majority of patients with advanced cancer (higher Gleason scores) had a lower PSA level (< 3 ng/mL) compared to those that had higher PSA levels (*p* value = 0.0001) [46]. This means that there are possibilities of being in advanced stage cancer but having very lower levels of PSA. This should guide clinicians mostly in resource‐limited areas who always rely on PSA outcome to refer patients for biopsy checkups. Bases on this, this practice could mislead them.

In the present study, comorbidities were very low among participants where only one participant had HIV (1/71, 1.41%), 6 had hypertension (6/71, 8.45%), one had diabetes (1/71, 1.41%), and one had dementia (1/71, 1.41%) and the largest number did not have any comorbidity (64/71, 90.14%). These results did not align well with those of a retrospective study conducted by Katongole et al. [3], who found that 33 (3.78%) had cardiac disease, 49 (5.6%) had HIV, 56 (6.4%) had high blood sugar, and 154 (17.62%) had hypertension. The majority of patients with Pca (23/34, 67.65%) in this study had serum PSA values greater than 100 ng/mL. This result is in good agreement with Nzeyimana et al.’s findings [38] where PSA levels exceeding 200 ng/mL were seen in the majority of individuals (41/114, 36%). The current study’s results were consistent with those of Okolo et al. [42], where PSA levels > 100 ng/mL were seen in 37/67 (55.2%) cancer patients. It is important to note that, in the majority of cases, cancer is the primary suspect when PSA is more than 100 ng/mL, as opposed to other prostate issues.

The results of the current investigation, however, did not match well with those of Pudasaini et al. [34], who found that 6/11 adenocarcinoma patients had PSA levels below 90 ng/mL. The youthful age of the recruited participants (mean age of 63 ± 10.56 years) in comparison to the mean age of 74.20 ± 9.40 years in the current study may be the cause of this. However, several people (2/34) with Pca but with borderline PSA values (4–10 ng/mL) were found in this investigation. This result is comparable to that of Fatima et al., who found that six out of 44 patients had borderline serum PSA levels [17], which reported 6/44 patients with borderline serum PSA levels. The American Cancer Society states that men who have PSA values in the “borderline range,” which is between 4 and 10 ng/mL, have a one in four chance of having Pca [47]. This could be the cause of the correlation between several studies.

The majority of BPH participants in this study (20/36 or 55.56%) had serum PSA levels above 20.1 ng/mL, which is consistent with Josephine et al.’s findings [43] that many BPH cases showed serum PSA levels above 20 ng/mL. In the current study, only 4 out of 36 (11.11%) BPH subjects had serum PSA levels below the normal range (< 4 ng/mL). This result was similar to that of Jasani et al. [48], who observed that 19/81 (23.46%) of BPH cases had normal PSA levels, and Josephine et al., who discovered that many BPH cases had serum PSA levels below 4 ng/mL [43]. We observed total serum PSA values above 100 ng/mL in both BPH and Pca participants, which may be related to the possible underlying prostatitis in BPH participants which is inconsistent with what Samad et al. [35] reported that only prostate adenocarcinoma cases had levels above 100 ng/mL.

We also noted a significant correlation with total serum PSA levels < 4 ng/mL (*p* = 0.043) and 4.1–20.0 ng/mL (*p* = 0.010) with BPH and PSA levels > 100 ng/mL for prostate adenocarcinoma (*p* = 0.001). This result was comparable to that of Mathaiyan et al., [49] who found that patients who first reported with a UTI had a strong correlation (*p* < 0.0001) between higher blood PSA levels and Pca. The mean PSA levels in Pca and BPH were compared in this study, and the difference between the two means was statistically significant (*p* = 0.001; 55.88 ± 87.90 and 240.53 ± 167.09 ng/mL, respectively). This indicates that compared to those with adenocarcinoma, the serum PSA levels of BPH were noticeably lower. The results of this study were similar to those of a number of other studies, including Shetty and Shetty [50] and Pudasaini et al. [34], where the mean serum PSA levels for adenocarcinoma and BPH were 79.40 ± 52.78 ng/mL and 7.32 ± 5.74 ng/mL, respectively. According to Tindall et al. [51], there was a significant difference (*p* < 0.0001) in all PSA ranges between participants with Pca and those without.

The ROC curve analysis is a statistical test that helps in evaluating the diagnostic accuracy in tests that differentiate between diseased and nondiseased circumstances. With this curve, an AUC can be calculated which is a metric that summarizes the diagnostic performance of a test. According to Corbacioglu and Aksel, a biomarker with an AUC > 0.90 is excellent in diagnostic performance, good when its 0.80–0.89, and not good if its below 0.08 regardless of a good statistical significance and regarded a failure if its 0.5 and below [33]. Sensitivity of the test went on decreasing with an increase in PSA unlike specificity and PPV which increased with an increase in PSA threshold as they are clearly shown in Table 5.

Our ROC curve analysis showed an AUC of 0.0.85 (95% CI: 0.77–0.94), showing that the biomarker is good at differentiating for clinicians between malignant and benign lesions. Alden et al. reported an AUC of 0.624 using identical biomarkers. Compared to our optimal cut‐off point of 103.4 ng/mL, calculated by the highest Youden Index (J): 0.595, with a sensitivity of 68% and a specificity of 92%, which indicated a good predictive power portraying good clinical relevance, Alden et al. had optimal cut off of 8.6 ng/mL that was much lower because of the lower PSA thresholds they considered (2 ng/mL and 10 ng/mL) [52]. Nevertheless, we still see that their PPV and specificity also increased with increase in PSA and sensitivity decreased.

We however found out that some of the associations between serum PSA levels and prostate tissue histological findings were almost statistically significant (borderline values). For example, PIN, a precancerous lesion had a *p*‐value of 0.053 in the serum PSA range of 4.1 to 20 ng/mL, which is close to the threshold of 0.05 thus considered borderline. This could be due to a smaller sample size providing insufficient information to provide a conclusive confirmation which however prospects a relationship possibility. Similarly, a *p*‐value of 0.061 for BPH and Pca in the serum PSA range of 20.1 to 100 ng/mL is borderline too which could be suggesting a trend that could have been significant if the sample size was adequately a representative. There is need for clinical practitioners to interpret serum PSA results carefully putting into consideration other patient parameters like age, family history, prostate volume, PSA trend over time, any medications used, occupation and lifestyle, and comorbidity plus other diagnostic tests before making clinical decisions that could be beyond most peoples’ budget.

### 4.1. Strengths of the Study

This study’s sample size was appropriately determined, and its statistical power was satisfactory. This study adhered to accepted practices for reporting histology. We obtained precise and trustworthy PSA readings by using daily regular quality control methods. Calibration of the MAGLUMI fully‐auto chemiluminescence immunoassay machine (model: Maglumi 2000) was performed every 14 days to ensure accuracy and consistent outcomes. The accuracy and dependability of the results were guaranteed by the use of controls for the PSA measurement on Maglumi machine and stored cancer‐positive slides for histology.

### 4.2. Study’s Limitations

Although the statistical methods used were suitable for the data, this study had one major drawback which was the small sample size of 71 individuals. This limits its findings from being generalized to a larger population. Therefore, big sample‐sized studies are required to substantiate and expand these findings. Increasing the sample size would strengthen the results and make them more expounded and applicable to larger populations, which would lead to more accurate and reliable prostate diagnostic and therapeutic options in the region.

For PSA measurements, the MAGLUMI fully‐auto chemiluminescence immunoassay machine (model: Maglumi 2000) was unable to provide readings more than 400 ng/mL. Because of this, we were unable to compare our results with those of other studies that reported PSA levels exceeding 400 ng/mL [53].

## 5. Conclusion

There was a significant correlation between BPH with PSA levels up to 20 ng/mL and above 100 ng/mL for prostate adenocarcinoma. In some of the cases, however, total serum PSA levels were high for BPH and low for prostate adenocarcinoma. PSA test usefulness cannot be nullified but its accuracy and specificity have to be ascertained in order to increase its reliability. Future researches are argued to focus more on how to refine PSA‐based diagnostics through identifying any underlying unknown hereditary factors and probably better biomarkers that could be influencing PSA levels. With this, dependability increases and unnecessary biopsing reduces, thus alleviating anxiety in patients and probably their caregivers. The future studies should aim at including a larger and more diverse sample size to improve more on reliability and applicability of the results.

## Funding

No funding was received for this manuscript.

## Conflicts of Interest

The authors declare no conflicts of interest.

## Data Availability

The data that support the findings of this study are available from the corresponding author upon reasonable request. This is done for the sake of patients’ confidentiality and ethical restrictions.
